# Rapid prototyping modelling in oral and maxillofacial surgery: A two year retrospective study 

**DOI:** 10.4317/jced.52556

**Published:** 2015-12-01

**Authors:** Anni Suomalainen, Patricia Stoor, Karri Mesimäki, Risto K. Kontio

**Affiliations:** 1 Department of Radiology, University of Helsinki and HUS Radiology (Medical Imaging Center); 2 Department of Maxillofacial Surgery, Helsinki University Hospital, Helsinki, Finland

## Abstract

**Background:**

The use of rapid prototyping (RP) models in medicine to construct bony models is increasing.

**Material and Methods:**

The aim of the study was to evaluate retrospectively the indication for the use of RP models in oral and maxillofacial surgery at Helsinki University Central Hospital during 2009-2010. Also, the used computed tomography (CT) examination – multislice CT (MSCT) or cone beam CT (CBCT) - method was evaluated.

**Results:**

In total 114 RP models were fabricated for 102 patients. The mean age of the patients at the time of the production of the model was 50.4 years. The indications for the modelling included malignant lesions (29%), secondary reconstruction (25%), prosthodontic treatment (22%), orthognathic surgery or asymmetry (13%), benign lesions (8%), and TMJ disorders (4%). MSCT examination was used in 92 and CBCT examination in 22 cases. Most of the models (75%) were conventional hard tissue models. Models with colored tumour or other structure(s) of interest were ordered in 24%. Two out of the 114 models were soft tissue models.

**Conclusions:**

The main benefit of the models was in treatment planning and in connection with the production of pre-bent plates or custom made implants. The RP models both facilitate and improve treatment planning and intraoperative efficiency.

** Key words:**Rapid prototyping, radiology, computed tomography, cone beam computed tomography.

## Introduction

Rapid prototyping (RP) or additive manufacturing (AM) technologies can construct physical models from computer-aided design using three–dimensional (3D) printers (1). Techniques of RP were first introduced in the 1980s in the field of engineering; for a recent review see Goiato *et al.* (2). Mankovich *et al.* (3) initially reported the use of RP to construct models of bony structures. It is also possible to print soft tissue models and color the tumours and other structures of interest, for example nerves, arteries or veins in the models. Most commonly used RP technologies for medical applications are stereolithography and 3D printing (3DP) (1). 3DP is advantageous to stereolithography for better accuracy, faster printing, and lower cost (1).

Better availability, shorter processing time, and descending costs have resulted in the increased use of RP. Concomitantly the development of medical applications is expanding. Five main groups in current applications are distinguished: 1) preoperative planning, surgical training and teaching, 2) manufacturing of inert implants, 3) production of surgical instruments and special equipment associated with the operations, 4) manufacturing of postoperative guides, long-term supports and aids, and 5) production of artificial tissue (4). The first four of these are already in general use, and the last one is under continuous investigation.

CT is by far the most used imaging modality for RP, but also MRI, 3D ultrasonography and nuclear isotope imaging have been used for this indication (5). Nowadays cone beam CT (CBCT) imaging offers an alternative imaging method for modeling (6). RP models have been used in different medical areas such as maxillofacial and craniofacial surgery, implantology, neurosurgery, orthopedics, scaffolds of ceramic, polymeric, and metallic materials, and fabrication of personalized maxillofacial prostheses (2). The accuracy of RP models corresponds to that of physical models with acceptable precision (7) and the different indications for RP modelling have an impact on the quality needed (5). For example, implant design usually requires highly accurate modelling, whereas the fabrication of models for preoperative planning or training might need less accuracy (5).

The aim of the present study was to evaluate retrospectively the use of RP models in oral and maxillofacial surgery in Department of Maxillofacial Surgery, Helsinki University Hospital, Helsinki, Finland during a two year period, with the focus set on the indications for their use and the CT examination method used for the acquisition of the data needed for rapid prototyping.

The research protocol was approved by the Ethics Committee of Helsinki University Central Hospital.

## Material and Methods

Based on the documented orders to the manufacturer of the RP models, we evaluated retrospectively the indications for the use of all RP models in oral and maxillofacial surgery at the Helsinki University Central Hospital during 2009-2010.

Patient data sets obtained by multislice computed tomography (MSCT; GE BrightSpeed 16, General Electric Medical Systems, Milwaukee, WI) or cone beam computed tomography (CBCT; Promax3D, Planmeca, Helsinki, Finland) were stored in Digital Imaging and Communications in Medicine (DICOM) format on a CD-Rom disc and sent to a commercial RP model manufacturer (Planmeca, Helsinki, Finland). From the data the Planmeca ProModel system manufactures directly an anatomical model utilizing layer by layer 3D printing.

In addition to conventional hard tissue models, also special models were fabricated. The special models included soft tissue models or models with colored tumour or other structure(s) of interest. For the fabrication of the colored models the tumour or other structure(s) of interest was indicated with arrows in the JPEG images of the CT examinations, which were included in the data sent to the manufacturer.

## Results

During the study period in total 114 RP models for 102 patients were fabricated, 52 models in 2009 and 62 in 2010. The treatment of one patient required four models, two patients needed three models, and for five patients two models were fabricated. The mean age of the patients and the number of models is presented in  Table 1.

**Table 1:**
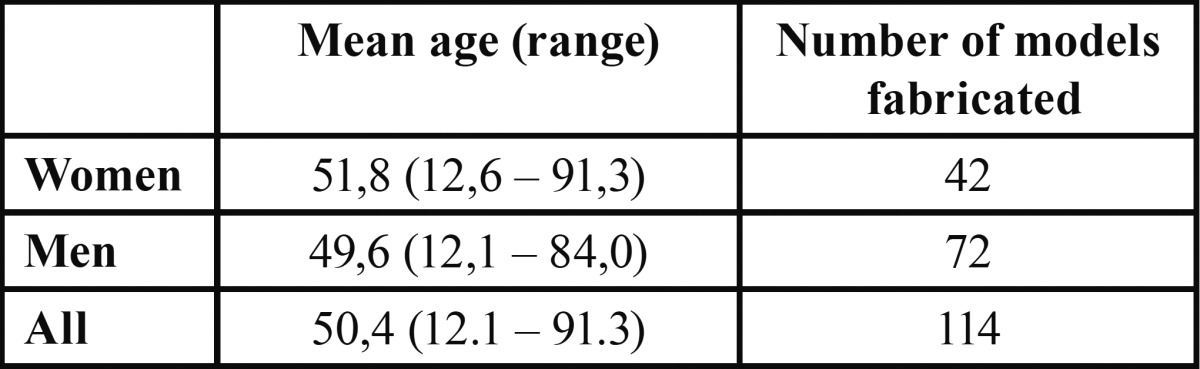
The mean age of the patients and the number of models.

The number of the models according to the indication for their production, and the number of special models are presented in  Table 2. The main indication for the modelling was malignant lesions in 29% and secondary reconstructions in 25%. Most of the models, 75%, were conventional models of hard tissues. Color was used in every fourth model for visualization of tumour or other structure(s) of interest. Two of the models presented solely soft tissues. Six models were produced from another part than the facial region of the body, i.e. arm (2), tibia (2), femur and scapula.

**Table 2:**
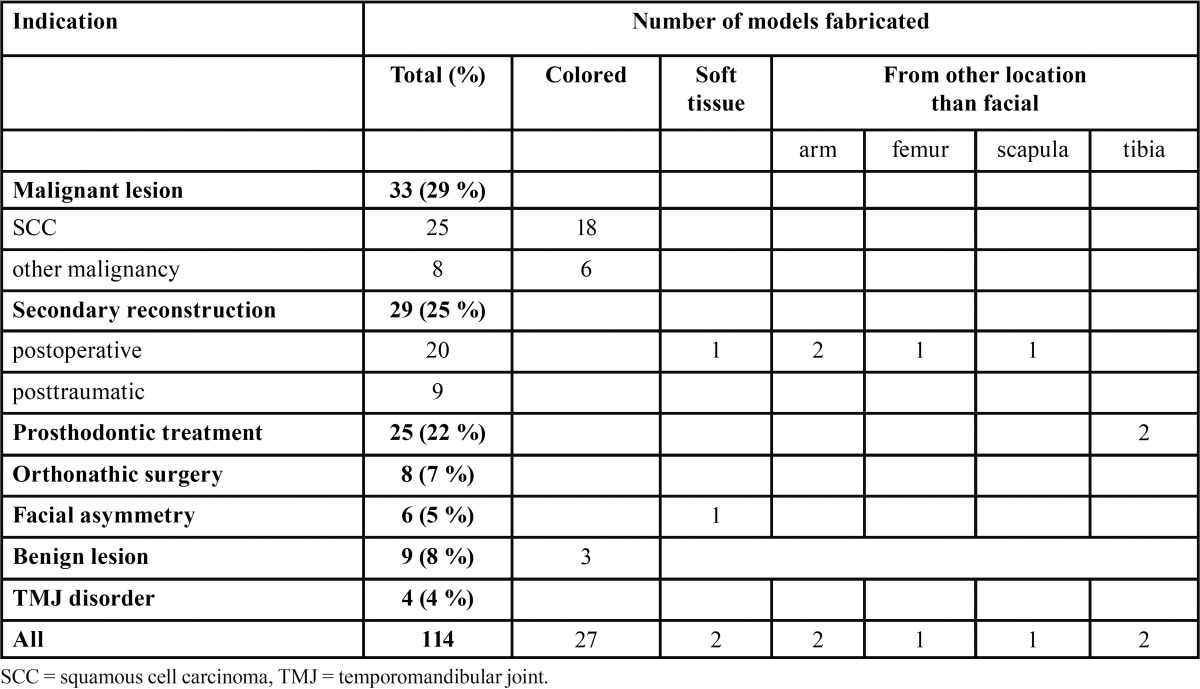
Number of fabricated rapid prototyping models, their special features and extra facial location, according to the indication of modelling.

For the acquisition of data, MSCT was used in 81% and CBCT in 19% of the cases ( Table 3).

**Table 3:**
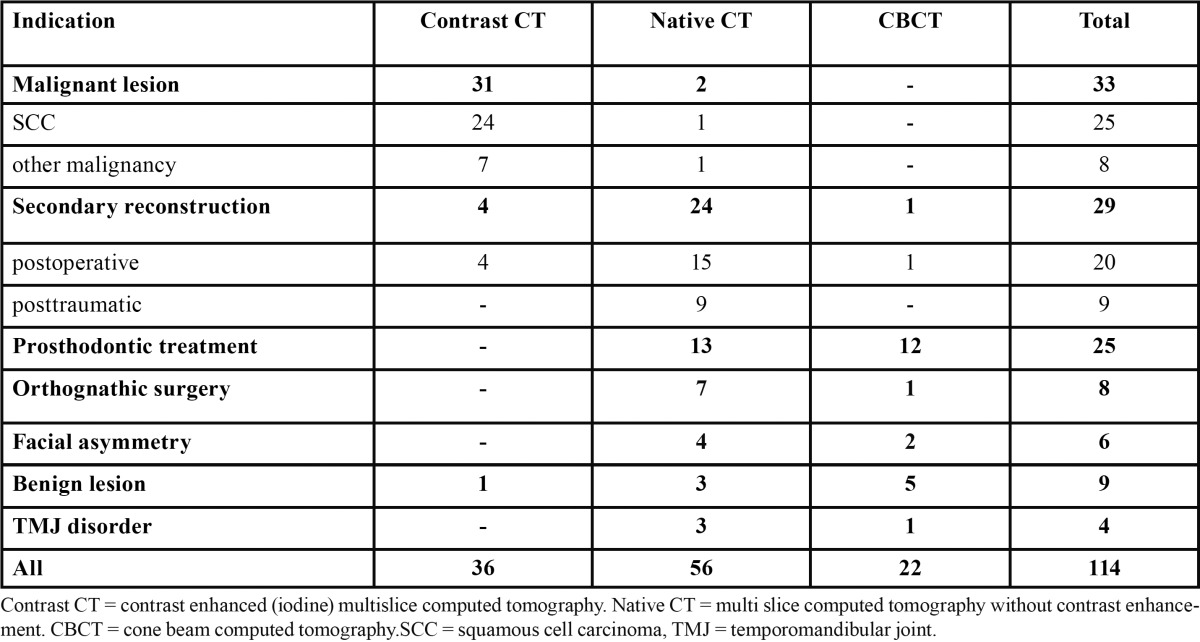
Type of CT examination used for acquisition of data for rapid prototype modelling according to the indication.

-Malignant lesions

**Figure 1:**
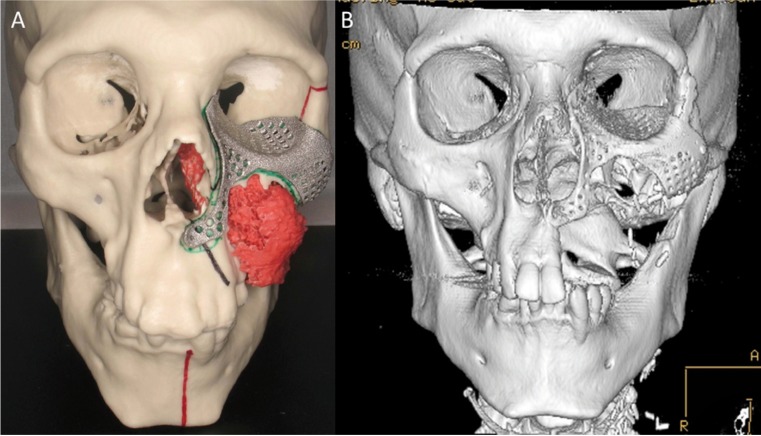
A) In this model the fibrosarcoma of the maxillar region is marked with red color. The RP model was used for treatment planning. CT data was also used to design 3D volumetric virtual implant. The shape of the implant was designed using the mirroring technique of the opposite intact orbit. B) Postoperative 3D MSCT image of the patient.

In case of a malignant lesion ( Table 2, Table 4) coloring of the tumour tissue was done in 73% (24/33) of the models in order to better visualize its location and to be able to plan the extent of the resection in detail (Fig. 1) ( Table 4). In two out of the 31 operated patients the resection marginal was not tumour-free. In one the marginal was negative in the orbital region. However, the patient has been tumour free after the treatment consisting of surgical treatment and chemoradiation therapy. The other patient had a metastasis from renal carcinoma in the maxilla and the indication for the treatment was not curative. In addition to treatment planning, the models were used to plan custom made surgical guides, pre-bent plates and custom made implants (Fig. 1) ( Table 4).

**Table 4:**
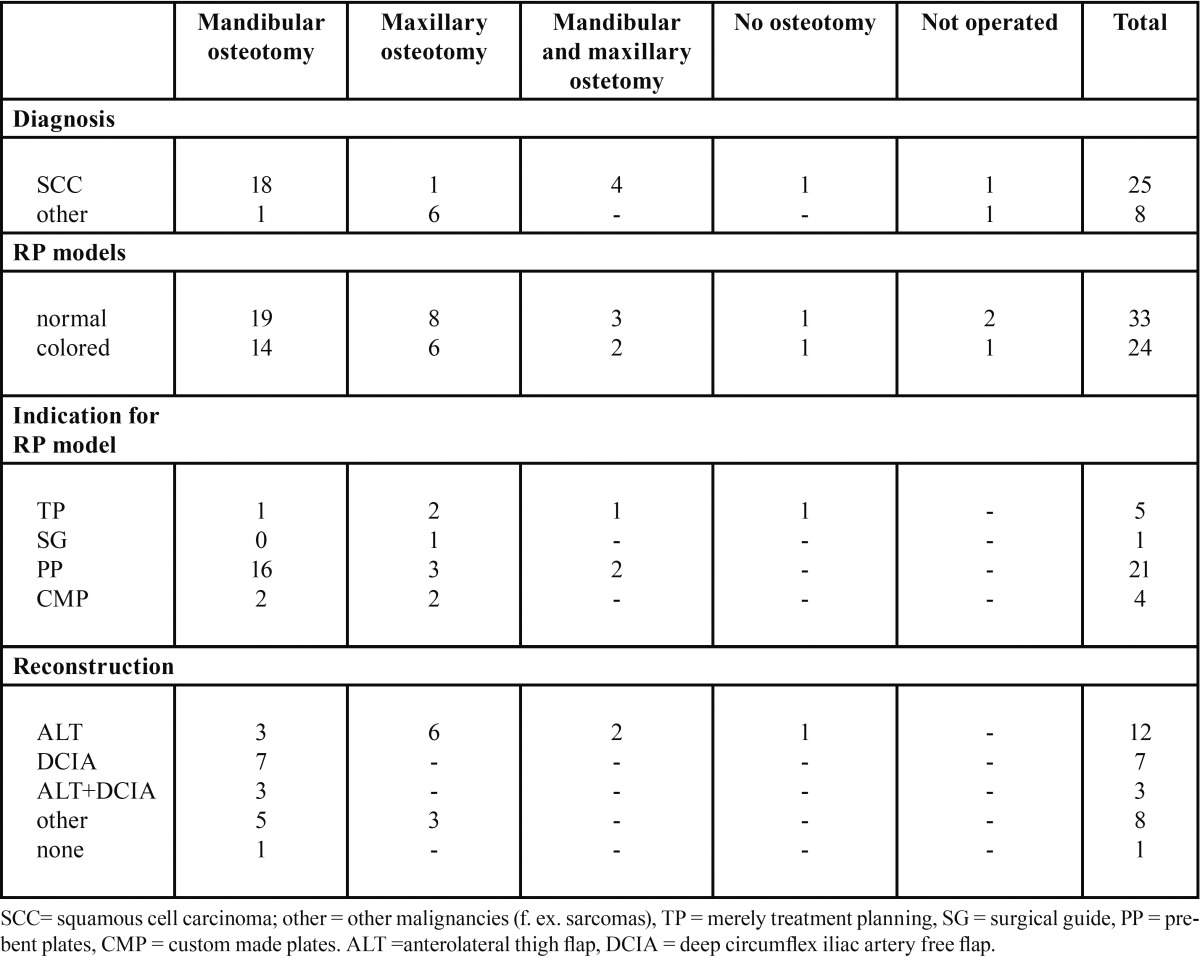
Treatment procedures of the patients with malignancies according to diagnosis, indication for modelling, type of model, and type of reconstruction.

-Secondary reconstruction

a. Postoperative reconstruction 

For 14 patients 20 models were fabricated for postoperative reconstruction.

They included 9 carcinoma or sarcoma patients, 3 patients with large odontogenic cysts or tumours, one with a vascular malformation, and one lymphoma patient, who had lost the maxilla because of a severe fungal infection.

b. Posttraumatic reconstruction

Nine models were produced for 8 patients needing a posttraumatic reconstruction.

In three patients a secondary reconstruction of an orbital fracture was done with custom made titanium implants. In one patient a good functional and aesthetic result and in two patients obvious improvement was achieved.

In one patient suffering from posttraumatic ankylosis of the TMJ two RP models were ordered.

In four patients the RP models were used for surgical planning, but three of them refused surgery. In the fourth patient a persistent oroantral fistula was surgically closed.

-Prosthodontic treatment

Prosthodontic treatment was the indication for 25 models in 22 patients (22% of the cases).

The indications consisted of postoperative treatment planning (7 patients), severe hypoplasia of the maxilla (6 patients), cleft palate (4), posttraumatic condition (3), severe periodontitis and total loss of teeth (1), and ectodermal dysplasia (1).

The patients have been exposed to extensive surgical treatment including tissue augmentation procedures. Hitherto, 14 patients have obtained implant based rehabilitation of the occlusion with a fixed or removable denture. In one patient the treatment consisted merely of a sulcusplastia and a new fixed prosthesis. Seven patients have had no rehabilitative dental treatment yet.

-Orthognathic surgery or asymmetry

The indication for modelling in 14 patients was skeletal or soft tissue deformity which was combined in eight patients with malocclusion and in six patients with facial asymmetry.

**Figure 2:**
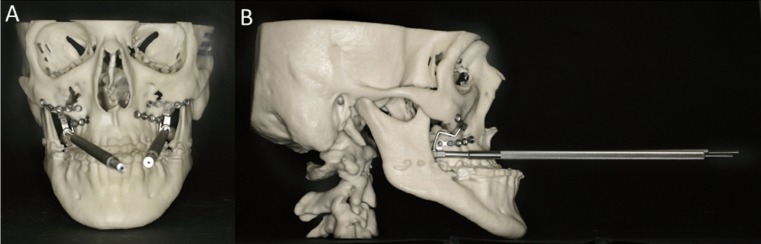
The RP model was fabricated for planning of distraction treatment (A) anteroposterior and B) lateral view).

In five of the eight malocclusion patients the models were used for planning of orthognaticbimaxillary osteotomies, in two for planning of distraction osteogenesis (Fig. 2) and in one patient for planning of genioplasty.

In one asymmetric patient the treatment was genioplasty. In a patient suffering from hemifacial microsomia a soft tissue model was manufactured and used for the soft tissue reconstruction, which was made with an ALT-flap. Prior to this the hemimandibular hard tissue deformity was reconstructed by a custom made TMJ prosthesis. For one patient lavation of the TMJ was the only treatment and for the remaining three patients diagnosed with asymmetry no surgery was performed.

-Benign lesions

Nine patients (8%) were diagnosed with a benign lesion.

**Figure 3:**
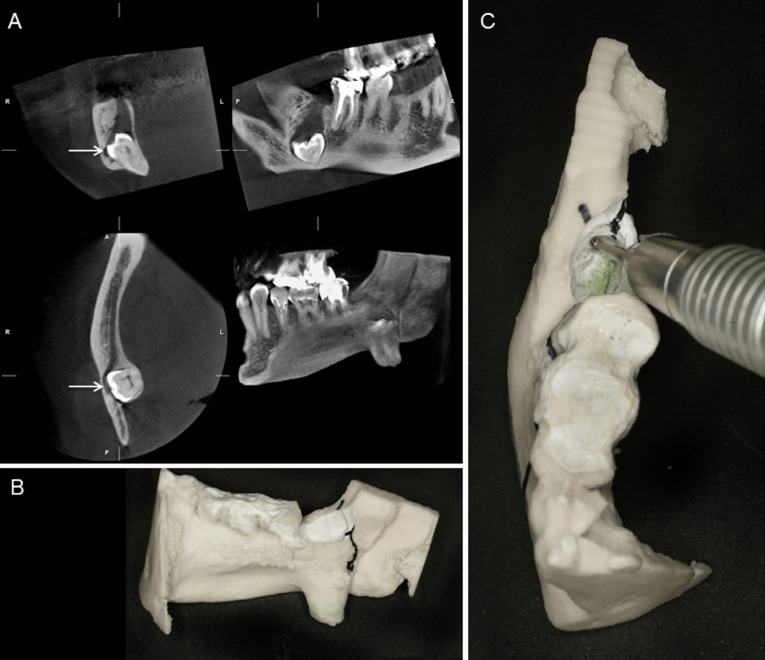
A) Follicular cyst of the right lower third molar has caused severe dislocation of the tooth. In the CBCT images the mandibular canal is marked with an arrow. B) and C) With the help of the model it was possible to plan the operation and it was used for surgical training.

They included actinomycosis, ossifying fibroma, odontogenic cyst, follicular cyst with dislocation of the lower third molar (Fig. 3), keratocystic odontogenic tumour, osteonecrosis, odontoma, pleomorphic adenoma, and reactive changes of the mandible. Except for the latter one, all patients underwent surgery.

The RP models were used for treatment planning and surgical training. In two patients the operation area was stabilized using custom made titanium plates.

-TMJ disorders

Four models were fabricated from the TMJ and adjacent structures. Two of the models were made of the same patient with severe arthrosis of the TMJs and restricted movements of the joints. In one patient a hyperplastic processus coronoideus interfered the opening of the mouth. One patient with juvenile arthritis leading to severely resorbed condyles was surgically treated with TMJ prostheses with good functional and aesthetic outcome.

## Discussion

The most abundant indication for fabricating the model in the present study was malignancy representing 29% of the cases. Simulation of the surgery can be performed and devices for osteosynthesis can be prefabricated with preoperative model planning saving operation theatre resources (8,9), improving patient outcome and reducing costs (10). In previous studies obvious reduced surgery time has been reported (11,12). With the help of the model the extent of the resection can be planned more exactly and when the tumour is colored in the model it´s easy to visualize its location and dimensions. In the present study this method was used in 73% of the models concerning malignancies.

A plate can be bent and adapted to fit the RP model or custom made plates can be ordered from the manufacturer based on the 3D-model data. The length of the plates and the number as well as location of the screws can be easily planned (9). Plate handling during surgery can thus be minimized which also preserves the strength of the plate (1). In 21 out of our 31 malignant tumour patient’s pre-bent plates were used, and additionally custom made implants were manufactured for four patients.

RP models can also be used for presurgical planning for reconstruction with vascularized or nonvascularized autogenous bone (1,10,13,14). Lethaus *et al.* (10) found this method feasible in routine clinical practice for microvascular reconstruction of the mandible.

Secondary reconstruction including postoperative and posttraumatic reconstruction was indication for the RP modelling in 25% in our study.

One indication for the use of the models with trauma patients is customized reconstruction of orbital wall defects using titanium mesh or sheet (15). The titanium mesh or sheet is shaped and sized to the required contours for implantation with mirror imaging of the contralateral side. The customized titanium implant accurately reproduces orbital contours and thus restores the orbital volume. This reduces operation theatre resources, improves the functional and aesthetic outcomes of post-traumatic orbital reconstruction and minimizes surgical complications (15,16). In the present study three patients had a secondary reconstruction of the orbital fracture with custom made titanium implants.

In one patient good functional and aesthetic result was achieved. However, in the other two patients the aesthetic result was not perfect even though improvement achieved. This highlights the difficulties, which are often confronted with orbital floor modelling (5,17) including adipose tissue atrophy and scar formation.

In the present study prosthodontics treatment was the indication for RP modelling in 22%. For prosthodontics treatment with implant based rehabilitation CBCT is frequently used nowadays. However, in the most severe cases with patients that have difficult anatomotophographic conditions this tool is not sufficient enough. In these cases the RP models can make the surgical planning easier. The functional occlusion and aesthetics of the dental implant treatment always depend on both jaws, the orthognathic relation between the mandibula and maxilla as well as the relation between the alveolar ridges and surrounding soft tissues. The 3D modelling facilitates the planning simply since all skeletal relations are clearly visible at once. Demanding planning for implant placement with need for augmentation and osteotomies can thus be visualized more clearly by using 3D models and modified treatment options for e.g. in patients with clefts or reconstruction with microvascular composite flaps can be planned safer with less risk of complication than planning based on (CB)CT examinations only. The final implant placement often needs guiding splints that can be manufactured based on the model and CT data as well.

In orthognatic surgery the use of virtual 3D planning is already frequently used. The indication for RP modelling in 13% of the patients was skeletal deformities combined with malocclusion and asymmetry in the present study. Our findings were in line with earlier observations showing that the modeling in orthognathic surgery increases intraoperative accuracy in patients with mandibular prognathism and/or maxillary retrusion (18). In addition to the orthognathic surgery planning, models can be used to reconstruct the templates to determine the size and shape of the bone grafts (19). We also found that with 3D model planning we could fit distractor devices successfully and very exactly during surgery reducing both risks for complications with the distraction axis and time needed in the surgical theatre.

The use of RP models and computer-assisted surgery is well established for complex craniofacial procedures as described earlier. These methods can also be used in the treatment of benign lesions (20). Stoetzer *et al.* (20) presented a case of a patient with a large follicular cyst of sinus maxillaris treated with computer-assisted surgery with RP procedures. After resection of the cyst, the sinus wall was reconstructed with a prebent 3D titanium-mesh using CAD/CAM technique. In the present study 8% of the patients had benign lesions.

In our study the surgeons could benefit the use of RP modelling also in patients with different TMJ disorders. 4% of the RP models were manufactured for this patient group. Two of the RP models were made for the same patient. TMJ disorder patients, with total resorption of the condyles can successfully be treated with TMJ prosthesis manufactured using 3D model data. This method is helpful in providing successful treatment with regard to symmetry of the face and stabile occlusion and improved function of the mandible simultaneously saving operation time (21). There are not yet available long follow-up periods of these patients but it has been shown that TMJ implant has provided normal jaw function for over 2 years after surgery (22).

Although the use of the RP modelling has many clinical applications and benefits as shown previously, the clinical problems are quite often nowadays solved using virtual 3D planning including for example virtual bending of reconstructive plates (13,23-25). However, RP models are still required in severe cases and time will show how the RP models are used in the future.

In conclusion, the RP models are used commonly in oral and maxillofacial surgery. Main indications for their use are preoperative planning, surgical training and teaching as well as the use of pre-bent plates and custom made implants. Improved treatment planning and intraoperative efficiency are obvious benefits for their use and compensate the inherent increase in costs.
